# Supplementary material for: Exploration of Potential Breath Biomarkers of Chronic Kidney Disease through Thermal Desorption–Gas Chromatography/Mass Spectrometry
Source: Metabolites. 2023 Jul 11;13(7):837. doi: 10.3390/metabo13070837 (PMC10385797; doi:10.3390/metabo13070837)
Supplement: Supplementary file 1 [file metabolites-13-00837-s001.zip › metabolites-2470805-supplementary.pdf]

Table S1. Data on the 324 volatile organic compounds (VOCs) that were measured in the breath of participants using TD–GCMS.

Variable	VOC_GROUP	VOC_NAME	Mol Weight (amu)	CAS Number
A_1	VSC	Methanethiol	48.003	000074-93-1
A_2	VSC	Dimethyl disulfide	93.991	000624-92-0
A_3	VSC	Dimethy sulfide	62.019	000075-18-3
A_4	VSC	Sulfur dioxide	63.962	007446-09-5
A_5	VSC	Carbon disulfide	75.944	000075-15-0
A_6	VSC	Methyl propenyl sulfide (E)	88.035	042848-06-6
A_7	VSC	Methyl propenyl sulfide (Z)	88.035	052195-40-1
A_8	VSC	Allyl methyl sulfide	88.035	010152-76-8
A_9	VSC	Methyl propyl sulfide	90.05	003877-15-4
A_10	VSC	Dimethyl sulfone	94.009	000067-71-0
A_11	VSC	Allyl Isothiocyanate	99.014	000057-06-7
A_12	VSC	Dimethyl Sulfoxide	78.014	000067-68-5

A_13	VSC	4-Methylaminophenol sulfate	221.0357	000055-55-0
A_14	VSC	Allyl sulfide	114.05	000592-88-1
A_15	VSC	2-Methyltetrahydrothiophene	102.05	001795-09-1
A_16	VSC	(2E)-2-butenyl methyl sulfide	102.05	032931-14-9
A_17	VSC	Allyl methyl disulfide	120.007	002179-58-0
A_18	VSC	2-Aminoethyl hydrogen sulfate	141.01	000926-39-6
A_19	VSC	2,4-Dimethylheptane	128.157	002213-23-2
A_20	VSC	3-Sulfo-L-alanine	169.005	000498-40-8
A_21	Ketones	Acetone	58.042	000067-64-1
A_22	Ketones	Methyl vinyl ketone	70.042	000078-94-4
A_23	Ketones	2-Butanone	72.058	000078-93-3
A_24	Ketones	2-Heptanone	114.104	000110-43-0
A_25	Ketones	3-Heptanone	114.104	000106-35-4
A_26	Ketones	2,3-Butanedione	86.037	000431-03-8

A_27	Ketones	Dihydro-2(3H)-furanone	86.037	000096-48-0
A_28	Ketones	2-Pentanone	86.073	000107-87-9
A_29	Ketones	Methyl isopropyl ketone	86.073	000563-80-4
A_30	Ketones	Acetoin	88.052	000513-86-0
A_31	Ketones	Cyclohexanone	98.073	000108-94-1
A_32	Ketones	Piperidinone	99.068	027154-43-4
A_33	Ketones	2-Methylpropyl methyl ketone	100.089	000108-10-1
A_34	Ketones	4-Heptanone	114.104	000123-19-3
A_35	Ketones	Acetophenone	120.058	000098-86-2
A_36	Ketones	D-Carvone	150.105	002244-16-8
A_37	Ketones	(+)-Isodihydoarvone	152.12	006909-25-7
A_38	Ketones	2-Isopropylidene-5-methylcyclohexanone	152.12	015932-80-6
A_39	Ketones	trans-Dihydrocarvone	152.12	005948-04-9
A_40	Ketones	Menthone	154.136	000089-80-5

A_41	Ketones	5-Methyl-2-(1-methylethyl)-, (2S-cis)-cyclohexanone	154.135	010458-14-7
A_42	Ketones	l-Menthone	154.136	014073-97-3
A_43	Ketones	6-Methylphenanthridine	193.089	003955-65-5
A_44	Ketones	2-Methyl-3-hexanone	114.104	007379-12-6
A_45	Ketones	(2R,5R)-2-Isopropyl-5-methylcyclohexanon	154.249	005146-52-1
A_46	Alcohol	Ethanol	46.042	000064-17-5
A_47	Alcohol	1-propanol	60.058	000071-23-8
A_48	Alcohol	2-Propanol	60.058	000067-63-0
A_49	Alcohol	1,2-Ethanediol	62.037	000107-21-1
A_50	Alcohol	Acetol	74.037	000116-09-6
A_51	Alcohol	1-Butanol	74.073	000071-36-3
A_52	Alcohol	2-Methyl-2-propanol	74.073	000075-65-0
A_53	Alcohol	2-Butanol	74.073	000078-92-2
A_54	Alcohol	2-Methylpropyl alcohol	74.073	000078-83-1

A_55	Alcohol	Isobutenylcarbinol	86.073	000763-32-6
A_56	Alcohol	3-Octanol	130.136	000589-98-0
A_57	Alcohol	3-Pentanol	88.089	000584-02-1
A_58	Alcohol	2-Methyl-2-butanol	88.089	000075-85-4
A_59	Alcohol	2,3-Butanediol	90.068	000513-85-9
A_60	Alcohol	1,3-Butanediol	90.068	000107-88-0
A_61	Alcohol	1-Methoxy-2-propanol	90.068	000107-98-2
A_62	Alcohol	Phenol	94.042	000108-95-2
A_63	Alcohol	3-Methoxybutanol	104.084	002517-43-3
A_64	Alcohol	1,2-Propanediol	106.063	000623-39-2
A_65	Alcohol	Benzenemethanol	108.058	000100-51-6
A_66	Alcohol	2,3-Dimethyl-2,3-butanediol	118.099	000076-09-5
A_67	Alcohol	2-Ethyl-1-Hexanol	130.136	000104-76-7
A_68	Alcohol	2-Phenyl-2-propanol	136.089	000617-94-7

A_69	Alcohol	2-Phenoxy-ethanol	138.068	000122-99-6
A_70	Alcohol	(2,5-Dimethyltetrahydro-2H-pyran-2-yl)methanol	144.115	054004-46-5
A_71	Alcohol	2,6-Dimethyl-4-heptanol	144.151	000108-82-7
A_72	Alcohol	2,5-Dimethyl-2,5-hexanediol	146.131	000110-03-2
A_73	Alcohol	2-(4-Methyl-3-cyclohexen-1-yl)-2-propanol	154.136	000098-55-5
A_74	Alcohol	Menthol	156.151	001490-04-6
A_75	Alcohol	(+-)-Isomenthol	156.151	023283-97-8
A_76	Alcohol	Cis-1,3,trans-1,4-Menthol	156.151	000089-78-1
A_77	Alcohol	Trans-1,3,trans-1,4-Menthol	156.151	000491-01-0
A_78	Alcohol	L-(-)-Menthol	156.151	002216-51-5
A_79	Alcohol	Menthol, (.+-.)-	156.151	015356-70-4
A_80	Alcohol	3-Allyl-2-methoxyphenol	164.084	001941-12-4
A_81	Alcohol	4-Nonylphenol	220.183	000104-40-5
A_82	Alcohol	(S)-1,3-Butanediol	90.068	024621-61-2

A_83	Alcohol	2-Butoxy-ethanol	118.17	000111-76-2
A_84	Alcohol	Cyclohexanol	100.089	000108-93-0
A_85	Alcohol	2-(2-Ethoxyethoxy)-ethanol	134.094	000111-90-0
A_86	Alcohol	2-Methyldecane	156.31	006975-98-0
A_87	Alcohol	1-(1,5-Dimethylhexyl)-4-methylcyclohexanol	226.23	024945-44-6
A_88	Alcohol	3,7-Dimethyl-1,6-octadien-3-ol	154.136	000078-70-6
A_89	Halo-Hydrocarbons	Dichloroethene	95.953	000156-59-2
A_90	Halo-Hydrocarbons	Ethane, 1,1-difluoro-	66.028	000075-37-6
A_91	Halo-Hydrocarbons	Dichloro-, radical ion(1+) methane	83.953	058165-12-1
A_92	Halo-Hydrocarbons	Methylene chloride	83.953	000075-09-2
A_93	Halo-Hydrocarbons	1,2-Dichloro- ethane	97.969	000107-06-2

A_94	Halo-Hydrocarbons	1-Chloro-1,1-difluoro- ethane	99.989	000075-68-3
A_95	Halo-Hydrocarbons	1,2-Dichloro- propane	111.985	000078-87-5
A_96	Halo-Hydrocarbons	Chloroform	117.914	000067-66-3
A_97	Halo-Hydrocarbons	(Z)-1,2-Dichloro- ethylene	152.12	000076-22-2
A_98	Halo-Hydrocarbons	Bromodichloro- methane	161.864	000075-27-4
A_99	Halo-Hydrocarbons	(E)-1,2-Dichloroethylene	95.953	000156-60-5
A_100	Halo-Hydrocarbons	Trichloroethene	129.914	000079-01-6
A_101	Halo-Hydrocarbons	Dibromo(chloro)methane	205.813	000124-48-1
A_102	Halo-Hydrocarbons	Iodomethane	141.928	000074-88-4

A_103	Halo-Hydrocarbons	Tetrachloroethene	163.876	000127-18-4
A_104	Halo-Hydrocarbons	Chlorobenzene	112.008	000108-90-7
A_105	Alkenes	1,2-Pentadiene	68.063	000591-95-7
A_106	Alkenes	3-Methyl-1,2-butadiene	68.063	000598-25-4
A_107	Alkenes	2-Methyl-1,3-cyclopentadiene	80.063	003727-31-9
A_108	Alkenes	1-Pentene	70.078	000109-67-1
A_109	Alkenes	2-Methyl-1-pentene	84.094	000763-29-1
A_110	Alkenes	4-Methyl-1-pentene	84.094	000691-37-2
A_111	Alkenes	1-Hexene	84.094	000592-41-6
A_112	Alkenes	(3Z)-3-Methyl-3-hexene	98.11	004914-89-0
A_113	Alkenes	3-Methyl-3-hexene	98.11	000692-24-0
A_114	Alkenes	3-Methylene-1-vinylcyclopentene	106.078	061142-07-2
A_115	Alkenes	4-Methyl-3-heptene	112.125	004485-16-9

A_116	Alkenes	1-Octene	112.125	000111-66-0
A_117	Alkenes	2,4-dimethyl-1-heptene	126.141	019549-87-2
A_118	Alkyne	2-Pentyne	68.063	000627-21-4
A_119	Alkane	1,1-Diethoxyethane	118.099	000105-57-7
A_120	Alkane	3-Ethyl-5-(2-ethylbutyl)octadecane	366.423	055282-12-7
A_121	Alkane	3-Methylnonane	142.172	005911-04-6
A_122	Alkane	Dimethylmethane	44.063	000074-98-6
A_123	Alkane	Butane	58.078	000106-97-8
A_124	Alkane	Methylcyclobutane	70.078	000598-61-8
A_125	Alkane	Diethyl ether	74.073	000060-29-7
A_126	Alkane	Cyclohexane	84.094	000110-82-7
A_127	Alkane	1,2,4-trimethylcyclohexane	126.141	002234-75-5
A_128	Alkane	4-Methyl nonane	142.172	017301-94-9
A_129	Alkane	2,3-Dimethylbutane	86.11	000079-29-8

A_130	Alkane	2-Methyl pentane	86.11	000107-83-5
A_131	Alkane	Hexane	86.11	000110-54-3
A_132	Alkane	Methylcyclohexane	98.11	000108-87-2
A_133	Alkane	2,4-Dimethylpentane	100.125	000108-08-7
A_134	Alkane	Heptane	100.125	000142-82-5
A_135	Alkane	3-Methyl-hexane	100.125	000589-34-4
A_136	Alkane	2-Methylhexane	100.125	000591-76-4
A_137	Alkane	Cyclopentane	70.078	000287-92-3
A_138	Alkane	Propylcyclopentane	112.125	002040-96-2
A_139	Alkane	Ethylcyclohexane	112.125	001678-91-7
A_140	Alkane	4-Methylheptane	114.141	000589-53-7
A_141	Alkane	2-Methylheptane	114.141	000592-27-8
A_142	Alkane	Octane	114.141	000111-65-9
A_143	Alkane	1,2-Diethoxyethane	118.099	000629-14-1

A_144	Alkane	2,2,3,3-Tetramethylpentane	128.157	007154-79-2
A_145	Alkane	2,3,4-Trimethylhexane	128.157	000921-47-1
A_146	Alkane	4-Methyloctane	128.157	002216-34-4
A_147	Alkane	n-Nonane	128.157	000111-84-2
A_148	Alkane	3,5-Dimethyloctane	142.172	015869-93-9
A_149	Alkane	2-Methylnonane	142.172	000871-83-0
A_150	Alkane	2,6-Dimethyloctane	142.172	002051-30-1
A_151	Alkane	2,5-Dimethyloctane	142.172	015869-89-3
A_152	Alkane	Decane	142.172	000124-18-5
A_153	Alkane	5-Ethyl-2-methyl- octane	156.188	062016-18-6
A_154	Alkane	Undecane	156.188	001120-21-4
A_155	Alkane	3,7-Dimethyl-decane	170.204	017312-54-8
A_156	Alkane	5-Methylundecane	170.204	001632-70-8
A_157	Alkane	4-Ethyldecane	170.204	001636-44-8

A_158	Alkane	3,6-Dimethyldecane	170.204	017312-53-7
A_159	Alkane	Heptane, 2,2,4,6,6-pentamethyl-	170.204	013475-82-6
A_160	Alkane	Dodecane	170.204	000112-40-3
A_161	Alkane	2,6,7-Trimethyldecane	184.219	062108-25-2
A_162	Alkane	2,6,6-Trimethyl-decane	184.219	062108-24-1
A_163	Alkane	3,6-Dimethylundecane	184.219	017301-28-9
A_164	Alkane	Tridecane	184.219	000629-50-5
A_165	Alkane	3-Methyltridecane	198.235	006418-41-3
A_166	Alkane	Tetradecane	198.235	000629-59-4
A_167	Alkane	1,2,3,4,4a,5,8,9,12,12a-Decahydro-1,4-methanobenzocyclodecene	202.172	074708-73-9
A_168	Alkane	2,7,10-Trimethyldodecane	212.25	074645-98-0
A_169	Alkane	Pentadecane	212.25	000629-62-9
A_170	Alkane	2,6,11-Trimethyldodecane	212.25	031295-56-4
A_171	Alkane	2,6,10-Trimethyldodecane	212.25	003891-98-3

A_172	Alkane	Hexadecan	226.266	000544-76-3
A_173	Alkane	2,2,3,3,6,8,8-heptamethylnonane	226.44	*(CID21107818)
A_174	Alkane	2,2,11,11-Tetramethyldodecane	226.266	127204-12-0
A_175	Alkane	2,2,4,4,6,8,8-Heptamethylnonane	226.266	004390-04-9
A_176	Alkane	8-Methylheptadecane	254.297	013287-23-5
A_177	Alkane	Octadecane	254.297	000593-45-3
A_178	Alkane	Nonadecane	268.313	000629-92-5
A_179	Alkane	Icosane	282.329	000112-95-8
A_180	Alkane	2,6,10,14-Tetramethylhexadecane	282.329	000638-36-8
A_181	Alkane	Docosane	310.36	000629-97-0
A_182	Alkane	Isopentane	72.093	000078-78-4
A_183	Alkane	(2Z)-3-Methyl-2-hexene	98.11	010574-36-4
A_184	Alkane	Tetracosane	338.391	000646-31-1
A_185	Alkane	Heneicosane	296.344	000629-94-7

A_186	Alkane	Tricosane	324.376	000638-67-5
A_187	Terpenes	Isoprene	68.062	000078-79-5
A_188	Terpenes	3-methylene-6-(1-methylethyl)-cyclohexene	136.125	000555-10-2
A_189	Terpenes	p-Cymene	134.11	000099-87-6
A_190	Terpenes	Camphene	136.125	000079-92-5
A_191	Terpenes	(+)-Sabinene	136.125	003387-41-5
A_192	Terpenes	γ-Terpinene	136.125	000099-85-4
A_193	Terpenes	L-Limonene	136.125	005989-54-8
A_194	Terpenes	1-Methyl-4-(1-methylethylidene)-cyclohexene	136.125	000586-62-9
A_195	Terpenes	(R)-Limonene	136.125	005989-27-5
A_196	Terpenes	Myrcene	136.125	000123-35-3
A_197	Terpenes	3-Carene	136.125	013466-78-9
A_198	Terpenes	2-Pinene	136.125	000080-56-8
A_199	Terpenes	(+)-beta-Pinene	136.125	000127-91-3

A_200	Terpenes	(+)-Alpha-Pinene	136.125	007785-70-8
A_201	Terpenes	D-Sylvestrene	136.125	000499-03-6
A_202	Terpenes	(-)-Alpha-Pinene	136.125	007785-26-4
A_203	Terpenes	(-)-beta-Pinene	136.125	018172-67-3
A_204	Terpenes	1-Methyl-4-(1-methylethylidene)-cyclohexane	138.141	001124-27-2
A_205	Terpenes	1-Isobutylcyclohexene	138.141	003983-03-7
A_206	Terpenes	Bornane	138.141	000464-15-3
A_207	Terpenes	Carane	138.141	000554-59-6
A_208	Terpenes	Carane, (1S,3S,6R)-(-)-	138.141	002778-68-9
A_209	Aromatics	1,3,5-Trimethylbenzene	120.094	000108-67-8
A_210	Aromatics	o-Cymene	134.11	000527-84-4
A_211	Aromatics	m-Cymene	134.11	000535-77-3
A_212	Aromatics	Benzene	78.047	000071-43-2
A_213	Aromatics	Toluene	92.063	000108-88-3

A_214	Aromatics	Benzoic acid	122.037	000065-85-0
A_215	Aromatics	Ethylbenzene	106.078	000100-41-4
A_216	Aromatics	Styrene	104.063	000100-42-5
A_217	Aromatics	(2-Methyl-1-propenyl)benzene	132.094	000768-49-0
A_218	Aromatics	2-Butenyl-benzene	132.094	001560-06-1
A_219	Aromatics	p-Xylene	106.078	000106-42-3
A_220	Aromatics	Xylene	106.078	001330-20-7
A_221	Aromatics	o-Xylene	106.078	000095-47-6
A_222	Aromatics	m-Xylene	106.078	000108-38-3
A_223	Aromatics	(1-Methylethyl)-benzene	120.094	000098-82-8
A_224	Aromatics	Alpha-methylstyrene	118.078	000098-83-9
A_225	Aromatics	1-Ethyl-3-methylbenzene	120.094	000620-14-4
A_226	Aromatics	1,2,3-Trimethylbenzene	120.094	000526-73-8
A_227	Aromatics	Naphthalene	128.063	000091-20-3

A_228	Aromatics	Azulene	128.063	000275-51-4
A_229	Aromatics	1,2,3,5-tetramethylbenzene	134.11	000527-53-7
A_230	Aromatics	2-Ethyl-1,4-dimethylbenzene	134.11	001758-88-9
A_231	Aromatics	4-Ethyl-1,2-dimethylbenzene	134.11	000934-80-5
A_232	Aromatics	1-Ethyl-2,3-dimethylbenzene	134.11	000933-98-2
A_233	Aromatics	Phthalic anhydride	148.016	000085-44-9
A_234	Aromatics	1-Methoxy-4-[(1E)-1-propen-1-yl]benzene	148.089	000104-46-1
A_235	Acetate	Acetic acid, methyl ester	74.037	000079-20-9
A_236	Acetate	Formic acid, ethyl ester	74.037	000109-94-4
A_237	Acetate	Acetic acid ethenyl ester	86.037	000108-05-4
A_238	Acetate	sopropenyl formate	86.037	032978-00-0
A_239	Acetate	Acetic acid ethyl ester	88.052	000141-78-6
A_240	Acetate	Acetic acid, butyl ester	116.084	000123-86-4
A_241	Acetate	Methyl 2-hydroxy-2-methylpropanoate	118.063	002110-78-3

A_242	Acetate	1-Butanol, 3-methyl-, acetate	130.099	000123-92-2
A_243	Acetate	Methyl 3-hydroxy-3-methylbutanoate	132.079	006149-45-7
A_244	Acetate	Ethyl 2-hydroxy-2-methylpropanoate	132.079	000080-55-7
A_245	Acetate	1,2,3-Propanetriol, monoacetate	134.058	000106-61-6
A_246	Acetate	Acetic acid, hexyl ester	144.115	000142-92-7
A_247	Acetate	Acetic acid, phenylmethyl ester	150.068	000140-11-4
A_248	Acetate	Methyl 2-hydroxybenzoate	152.047	000119-36-8
A_249	Acetate	2-Hydroxy-1,3-propanediyl diacetate	176.068	000105-70-4
A_250	Acetate	3-Hydroxy-1,2-propanediyl diacetate	176.068	025395-31-7
A_251	Acetate	2-Ethylhexyl acrylate	184.146	000103-11-7
A_252	Acetate	3,7-Dimethyl-1,6-octadien-3-yl acetate	196.146	000115-95-7
A_253	Acetate	Isobornyl acetate	196.146	000125-12-2
A_254	Acetate	2-Isopropyl-5-methylcyclohexyl acetate	198.162	002230-87-7
A_255	Acetate	3-Hydroxy-2,4,4-trimethylpentyl 2-methylpropanoate	216.173	074367-34-3

A_256	Acetate	1,2,3-Propanetriol, triacetate	218.079	000102-76-1
A_257	Acetate	Citronellylisovalerate	240.209	068922-10-1
A_258	Acetate	Isopropyl myristate	270.256	000110-27-0
A_259	Acetate	1,4-Dioxane	88.052	000123-91-1
A_260	Acid	Acetic acid	60.021	000064-19-7
A_261	Acid	Propanoic acid	74.037	000079-09-4
A_262	Acid	Butanoic acid	88.053	000107-92-6
A_263	Acid	2-Methylpropanoic acid	88.053	000079-31-2
A_264	Acid	3-Methylbutanoic acid	102.068	000503-74-2
A_265	Acid	2-Methylbutanoic acid	102.068	000116-53-0
A_266	Acid	Acetonic acid	104.047	000594-61-6
A_267	Acid	3-Methyl pentanoic acid	116.084	000105-43-1
A_268	Acid	(N-(-2-Acetamido))-2-aminoethanesulfonic acid	182.036	007365-82-4
A_269	Furan	(2R,5S)-2,5-Dimethyltetrahydrofuran	100.089	001003-38-9

A_270	Furan	2,2,5,5-Tetramethyltetrahydrofuran	128.12	015045-43-9
A_271	Furan	3,6-Dimethyl-4,5,6,7-tetrahydro-1-benzofuran	150.104	000494-90-6
A_272	Steroid	(14beta)-pregnane	288.282	016396-79-5
A_273	Aldehyde	Acetaldehyde	44.026	000075-07-0
A_274	Aldehyde	Methacrolein	70.042	000078-85-3
A_275	Aldehyde	2-Butenal	70.042	004170-30-3
A_276	Aldehyde	3-methyl-2-Butenal	84.058	000107-86-8
A_277	Aldehyde	2-Furaldehyde	96.021	000098-01-1
A_278	Aldehyde	Hexanal	100.089	000066-25-1
A_279	Aldehyde	Benzaldehyde	106.042	000100-52-7
A_280	Aldehyde	Heptanal	114.104	000111-71-7
A_281	Aldehyde	Octanal	128.12	000124-13-0
A_282	Aldehyde	Nonanal	142.136	000124-19-6
A_283	Aldehyde	(2Z)-3,7-Dimethyl-2,6-octadienal	152.12	000106-26-3

A_284	Aldehyde	Decanal	156.151	000112-31-2
A_285	Other	Dimethylselenide	109.963	000593-79-3
A_286	Other	Methyl isopropyl ether	74.073	000598-53-8
A_287	Nitrogen	Pyridine	79.042	000110-86-1
A_288	Nitrogen	Indole	117.058	000120-72-9
A_289	Nitrogen	Acetonitrile	41.027	000075-05-8
A_290	Nitrogen	1,2-Dimethylhydrazine	60.069	000540-73-8
A_291	Nitrogen	Nitromethane	61.016	000075-52-5
A_292	Nitrogen	1-(1-Pyrrolidinyl)-1-icosanone	365.366	*(ChemSpider ID:29760013)
A_293	Nitrogen	1-(1-Pyrrolidinyl)-1-docosanone	393.397	*(ChemSpider ID:29760063)
A_294	Other	Ethylene oxide	44.026	000075-21-8
A_295	Other	1-Methoxy-2-methylpropane	88.089	000625-44-5
A_296	Other	(1S)-1,7,7-Trimethylbicyclo[2.2.1]heptan-2-one	152.12	000464-48-2

A_297	Other	1,8-Cineol	154.136	000470-82-6
A_298	Other	n-Heptyl methylphosphonofluoridate	196.103	162085-82-7
A_299	Other	Sevoflurane	200.007	028523-86-6
A_300	Other	4-[5-(2-Methoxyphenyl)-1,3,4-oxadiazol-2-yl]pyridine	253.085	*(CID706995)
A_301	Other	Cyclohexanone, 2-methyl-5-(1-methylethenyl)-, trans-	152.12	005524-05-0
A_302	Other	Propanethioic acid, 2-methyl-, S-ethyl ester	132.061	002432-50-0
A_303	Other	Thiosulfuric acid S-[2-[[5-[O-tolyloxy]pentyl]amino]propyl ester	347.123	*(CID548547)
Excluded	Siloxane	Trimethylsilanol	90.05	001066-40-6
Excluded	Siloxane	Dimethyl(1,1,2-trimethylpropyl)silanol	160.128	055644-10-5
Excluded	Siloxane	Benzeneethanamine, N-[(pentafluorophenyl)methylene]-.beta.,4-bis[(trimethylsilyl)oxy]	475.6	055429-85-1
Excluded	Siloxane	5,5,10,10-Tetramethyl-5,10-dihydrosilanthrene	268.11	033022-24-1
Excluded	Siloxane	Trimethyl(2-trimethylsilyloxyethylsulfanyl)silane	222.093	078921-31-0

Excluded	Siloxane	Methyl [(trimethylsilyl)oxy](4-[(trimethylsilyl)oxy]phenyl)acetate	326.137	055334-40-2
Excluded	Siloxane	Benzeneacetic acid, .alpha.,4-bis[(trimethylsilyl)oxy]-, trimethylsilyl ester	384.161	037148-64-4
Excluded	Siloxane	alpha,4-Bis[(trimethylsilyl)oxy]benzeneacetic acid ethyl ester	340.153	*(CID622432)
Excluded	Siloxane	m-Hydroxymandelic acid, tris(trimethylsilyl)-	384.161	068595-69-7
Excluded	Siloxane	Difluoro(dimethyl)silane	96.02	000353-66-2
Excluded	Siloxane	Hexamethyl disiloxane	162.09	000107-46-0
Excluded	Siloxane	1,1,3,3,5,5-hexamethyltrisiloxane	208.077	001189-93-1
Excluded	Siloxane	2,2,4,4,6,6-Hexamethyl-1,3,5,2,4,6-trioxatrisilinane	222.056	000541-05-9
Excluded	Siloxane	2,4,6,8-tetramethyl-1,3,5,7,2,4,6,8-tetraoxatetrasilocane	240.013	002370-88-9
Excluded	Siloxane	2,2,4,4,6,6,8,8-Octamethyl-1,3,5,7,2,4,6,8-tetroxatetrasilocane	296.075	000556-67-2
Excluded	Siloxane	Decamethyltetrasiloxane	310.127	000141-62-8
Excluded	Siloxane	Methyltris(trimethylsiloxy)silane	310.127	017928-28-8

Excluded	Siloxane	3,3-Diisopropoxy-1,1,1,5,5,5-hexamethyltrisiloxane	324.161	018082-56-9
Excluded	Siloxane	1-(4-Trimethylsilyloxyphenyl)-2-trimethylsilylaminoethanol trimethylsilyl ether	339.169	*(CID91739849)
Excluded	Siloxane	1,1,3,3,5,5,7,7,9,9-Decamethylpentasiloxane	356.115	000995-83-5
Excluded	Siloxane	p-Trimethylsilyloxyphenyl-bis(trimethylsilyloxy)ethane	370.182	*(CID622436)

Abbreviations: VSC, volatile sulfur compound; TD–GCMS, thermal desorption–gas chromatography/mass spectrometry.

Table S2. The fifty-eight breath volatile organic compounds (VOCs) and two clinical variables that were significantly different between patients with chronic kidney disease (non-dialysis CKD + hemodialysis) and normal healthy control groups as determined via *t*-test.

INDEP_VAR_NAME	*p*-value	EQ_VAR_PVAL	INDEP_VAR
Cyclohexanone	0.0000	0.0010	A_31
Acetophenone	0.0000	0.0238	A_35
1,2-Dichloro-ethane	0.0000	0.0008	A_93
Heptane	0.0000	0.9624	A_134
Decane	0.0000	0.3326	A_152
m-Xylene	0.0000	0.8969	A_222
Acetic acid ethyl ester	0.0000	0.0241	A_239
Nonanal	0.0000	0.0000	A_282
AGE	0.0001	0.0096	
Trichloroethene	0.0001	0.9999	A_100
n-Nonane	0.0004	0.1102	A_147

Sulfur dioxide	0.0006	0.0025	A_4
2-Methylpentane	0.0011	0.0219	A_130
p-Xylene	0.0011	0.0001	A_219
Azulene	0.0014	0.0001	A_228
3-Heptanone	0.0015	0.0040	A_25
4-Methylnonane	0.0016	0.0000	A_128
2-Ethyl-1-Hexanol	0.0021	0.0000	A_67
Systolic blood pressure	0.0030	0.2315	
Ethylcyclohexane	0.0032	0.0000	A_139
2-Hydroxy-1,3-propanediyl diacetate	0.0033	0.0000	A_249
Propylcyclopentane	0.0047	0.0000	A_138
Methanethiol	0.0049	0.0000	A_1
Nonadecane	0.0055	0.0000	A_178
3-Pentanol	0.0077	0.0000	A_57

3-Hydroxy-1,2-propanediyl diacetate	0.0082	0.0000	A_250
Menthol	0.0090	0.0000	A_74
Acetone	0.0102	0.2735	A_21
2-Butanone	0.0113	0.0170	A_23
Dihydro-2(3H)-furanone	0.0116	0.5507	A_27
Hexadecan	0.0116	0.0000	A_172
Phenol	0.0119	0.0000	A_62
Octanal	0.0134	0.4196	A_281
(1-Methylethyl)-benzene	0.0139	0.0108	A_223
4-Methylheptane	0.0158	0.0000	A_140
2-Butanol	0.0169	0.0000	A_53
(14beta)-pregnane	0.0180	0.0000	A_272
Ethylene oxide	0.0216	0.9437	A_294
3-Sulfo-L-alanine	0.0238	0.0361	A_20

2,5-Dimethyloctane	0.0280	0.0000	A_151
3-Methyl pentanoic acid	0.0281	0.0000	A_267
Pentadecane	0.0297	0.0000	A_169
(2R,5S)-2,5-Dimethyltetrahydrofuran	0.0297	0.0000	A_269
2-Phenoxy-ethanol	0.0316	0.0000	A_69
Allyl Isothiocyanate	0.0325	0.0000	A_11
Octadecane	0.0325	0.0000	A_177
Chloroform	0.0341	0.0000	A_96
Isoprene	0.0341	0.0000	A_187
Ethanol	0.0343	0.2734	A_46
2-Pentanone	0.0372	0.0000	A_28
2,4-dimethyl-1-heptene	0.0374	0.0000	A_117
3-Methylene-1-vinylcyclopentene	0.0391	0.0000	A_114
3-Carene	0.0423	0.0004	A_197

Methyl isopropyl ketone	0.0435	0.0000	A_29
2-Methylheptane	0.0439	0.3700	A_141
Acetic acid, butyl ester	0.0465	0.0106	A_240
Nitromethane	0.0477	0.0000	A_291
(Z)-1,2-Dichloro- ethylene	0.0485	0.0209	A_97
1-Methyl-4-(1-methylethylidene)- cyclohexane	0.0486	0.0000	A_204
(-)-beta-Pinene	0.0493	0.8214	A_203

Table S3. Training/test set analysis: 100 randomly repeated samplings (training vs. test set) and logistic regression multivariate modeling with the training set with an average accuracy of 0.714.

ITER NUM	Accuracy	CASE 0_0	CASE 0_1	CASE 1_0	CASE 1_1	AIC	MODEL
1	0.6923	4	1	3	5	10	A_130 + A_215 + A_222 + A_239
2	0.9167	3	1	0	8	14	AGE + A_187 + A_100 + A_134 + A_178 + A_221
3	0.7692	4	1	2	6	10	AGE + A_31 + A_130 + A_239
4	0.5833	1	3	2	6	14	PE_SBP + A_21 + A_35 + A_187 + A_285 + A_177
5	0.7692	4	1	2	6	12	AGE + A_27 + A_67 + A_279 + A_299
6	0.5833	3	1	4	4	12	A_27 + A_93 + A_152 + A_215 + A_279
7	0.5385	2	3	3	5	12	AGE + A_31 + A_93 + A_142 + A_154
8	0.7500	3	1	2	6	8	AGE + A_152 + A_228
9	0.5385	2	3	3	5	10	AGE + PE_SBP + A_4 + A_177

10	0.8333	2	2	0	8	12	AGE + A_93 + A_100 + A_130 + A_196
11	0.4615	4	1	6	2	10	A_17 + A_222 + A_239 + A_281
12	0.8333	3	1	1	7	10	AGE + A_31 + A_147 + A_154
13	0.6154	1	4	1	7	12	A_35 + A_130 + A_178 + A_198 + A_213
14	0.7500	2	2	1	7	12	AGE + A_27 + A_209 + A_130 + A_299
15	0.6923	3	2	2	6	10	AGE + PE_SBP + A_4 + A_31
16	0.7500	3	1	2	6	10	A_35 + A_62 + A_130 + A_239
17	0.7692	3	2	1	7	14	A_25 + A_35 + A_130 + A_215 + A_222 + A_239
18	0.6364	2	1	3	5	14	AGE + PE_SBP + A_100 + A_147 + A_150 + A_215
19	0.6923	2	3	1	7	8	A_130 + A_134 + A_239
20	0.7273	3	0	3	5	10	AGE + PE_SBP + A_31 + A_130
21	0.9167	3	1	0	8	14	AGE + PE_SBP + A_62 + A_134 + A_154 + A_239

22	0.5000	1	3	3	5	8	AGE + PE_SBP + A_130
23	0.8462	5	0	2	6	14	AGE + A_31 + A_130 + A_134 + A_152 + A_239
24	0.6667	3	1	3	5	12	A_35 + A_93 + A_130 + A_239 + A_299
25	0.8462	5	0	2	6	12	AGE + A_31 + A_130 + A_215 + A_239
26	0.7500	3	1	2	6	8	AGE + PE_SBP + A_147
27	0.6923	3	2	2	6	10	AGE + PE_SBP + A_100 + A_130
28	0.7500	3	1	2	6	14	AGE + A_28 + A_187 + A_130 + A_134 + A_239
29	0.6923	3	2	2	6	12	AGE + A_31 + A_121 + A_134 + A_154
30	0.9167	4	0	1	7	16	A_25 + A_35 + A_130 + A_134 + A_141 + A_152 + A_239
31	0.6154	3	2	3	5	20	A_27 + A_28 + A_31 + A_25 + A_62 + A_93 + A_152 + A_222 + A_239
32	0.6667	2	2	2	6	12	AGE + A_31 + A_25 + A_121 + A_139

33	0.6154	3	2	3	5	10	A_31 + A_93 + A_130 + A_239
34	0.5833	2	2	3	5	10	AGE + A_31 + A_130 + A_215
35	0.6154	3	2	3	5	8	AGE + A_93 + A_134
36	0.5833	2	2	3	5	10	A_35 + A_130 + A_134 + A_152
37	0.5385	3	2	4	4	10	PE_SBP + A_150 + A_177 + A_281
38	0.7500	3	1	2	6	12	AGE + A_31 + A_134 + A_152 + A_239
39	0.5385	3	2	4	4	10	A_31 + A_35 + A_285 + A_281
40	0.5833	2	2	3	5	8	A_35 + A_130 + A_203
41	0.8462	4	1	1	7	10	AGE + PE_SBP + A_134 + A_239
42	0.9167	3	1	0	8	14	AGE + A_67 + A_130 + A_134 + A_152 + A_279
43	0.6923	2	3	1	7	10	AGE + A_27 + A_62 + A_130
44	0.5000	2	2	4	4	10	AGE + A_67 + A_130 + A_161

45	0.6923	2	3	1	7	10	AGE + A_62 + A_130 + A_142
46	0.9091	2	1	0	8	12	AGE + PE_SBP + A_67 + A_134 + A_239
47	0.9231	5	0	1	7	12	AGE + A_31 + A_134 + A_154 + A_239
48	0.8333	3	1	1	7	12	A_31 + A_130 + A_285 + A_177 + A_196
49	0.4615	2	3	4	4	12	A_4 + A_25 + A_203 + A_221 + A_222
50	0.7500	1	3	0	8	14	AGE + PE_SBP + A_35 + A_100 + A_134 + A_239
51	0.9167	3	1	0	8	14	AGE + PE_SBP + A_31 + A_134 + A_147 + A_239
52	0.6667	2	2	2	6	12	AGE + A_31 + A_130 + A_147 + A_154
53	0.6923	4	1	3	5	8	AGE + A_31 + A_130
54	0.9167	4	0	1	7	14	AGE + PE_SBP + A_31 + A_134 + A_147 + A_239
55	0.9091	2	1	0	8	14	AGE + PE_SBP + A_31 + A_152 + A_154 + A_198

56	0.8333	4	0	2	6	12	AGE + PE_SBP + A_67 + A_228 + A_239
57	0.7692	2	3	0	8	8	AGE + A_130 + A_134
58	0.9167	4	0	1	7	12	A_62 + A_130 + A_150 + A_177 + A_239
59	0.6923	3	2	2	6	10	A_147 + A_154 + A_213 + A_279
60	0.6667	2	2	2	6	12	A_35 + A_46 + A_93 + A_130 + A_222
61	0.7692	2	3	0	8	12	A_17 + A_25 + A_93 + A_215 + A_222
62	0.4167	0	4	3	5	12	A_62 + A_178 + A_222 + A_273 + A_281
63	0.6923	3	2	2	6	12	PE_SBP + A_121 + A_130 + A_141 + A_239
64	0.8182	1	2	0	8	10	AGE + PE_SBP + A_31 + A_239
65	0.6154	4	1	4	4	8	AGE + A_31 + A_299
66	0.5833	3	1	4	4	16	PE_SBP + A_28 + A_62 + A_93 + A_141 + A_177 + A_260

67	0.7273	1	2	1	7	12	PE_SBP + A_31 + A_134 + A_154 + A_239
68	0.5833	2	2	3	5	14	A_4 + A_31 + A_93 + A_134 + A_203 + A_20
69	0.6154	2	3	2	6	16	PE_SBP + A_27 + A_25 + A_35 + A_187 + A_178 + A_219
70	0.6667	2	2	2	6	10	AGE + A_35 + A_130 + A_299
71	0.8333	4	0	2	6	16	PE_SBP + A_23 + A_62 + A_100 + A_177 + A_197 + A_213
72	0.8333	4	0	2	6	14	AGE + A_31 + A_93 + A_100 + A_130 + A_178
73	0.6923	3	2	2	6	10	AGE + A_35 + A_130 + A_299
74	0.8333	3	1	1	7	14	AGE + A_100 + A_130 + A_177 + A_215 + A_222
75	0.7692	3	2	1	7	14	AGE + A_31 + A_121 + A_152 + A_154 + A_198
76	0.6667	4	0	4	4	12	AGE + PE_SBP + A_31 + A_25 + A_152
77	0.6667	1	3	1	7	12	PE_SBP + A_31 + A_25 + A_154 + A_178

78	0.8333	3	1	1	7	10	A_31 + A_130 + A_285 + A_177
79	0.8462	5	0	2	6	12	AGE + A_31 + A_25 + A_130 + A_215
80	0.8333	3	1	1	7	12	AGE + A_31 + A_215 + A_222 + A_239
81	0.6923	2	3	1	7	12	AGE + A_25 + A_35 + A_147 + A_239
82	0.9167	3	1	0	8	12	AGE + A_67 + A_100 + A_130 + A_134
83	0.3846	4	1	7	1	10	A_130 + A_177 + A_219 + A_281
84	0.5455	2	1	4	4	12	AGE + PE_SBP + A_31 + A_147 + A_222
85	0.8462	3	2	0	8	14	A_31 + A_35 + A_130 + A_134 + A_152 + A_239
86	0.5833	1	3	2	6	14	A_28 + A_35 + A_62 + A_169 + A_20 + A_299
87	0.7692	3	2	1	7	14	A_31 + A_40 + A_130 + A_215 + A_222 + A_239
88	0.8333	2	2	0	8	12	A_31 + A_130 + A_215 + A_222 + A_239

89	0.8333	3	1	1	7	12	AGE + PE_SBP + A_31 + A_100 + A_239
90	0.5833	1	3	2	6	16	PE_SBP + A_28 + A_121 + A_100 + A_134 + A_178 + A_233
91	0.5000	1	3	3	5	12	AGE + PE_SBP + A_141 + A_142 + A_279
92	0.5833	2	2	3	5	12	AGE + A_4 + A_130 + A_198 + A_222
93	0.7692	4	1	2	6	16	AGE + A_25 + A_35 + A_134 + A_215 + A_239 + A_299
94	0.7000	2	0	3	5	12	AGE + PE_SBP + A_134 + A_154 + A_239
95	0.7692	3	2	1	7	12	AGE + A_31 + A_130 + A_178 + A_196
96	0.5833	3	1	4	4	10	A_62 + A_130 + A_150 + A_239
97	0.7692	3	2	1	7	14	PE_SBP + A_16 + A_134 + A_141 + A_152 + A_239
98	0.8333	3	1	1	7	14	A_31 + A_130 + A_215 + A_222 + A_239 + A_299
99	0.8462	5	0	2	6	12	AGE + A_31 + A_93 + A_130 + A_239

100	0.6667	4	0	4	4	14	AGE + A_25 + A_215 + A_221 + A_222 + A_239

Abbreviations: AIC, Akaike information criterion.

Table S4. Significant continuous variables selected from the first step of multivariate analysis through one-hundred-times-repeated training and test sets. The variables are listed in order of frequency as shown in Table S3.

Name of VOC or clinical variable	VOC group	Frequency	VOC_variable
Age		60	
2-Methyl pentane	Alkane	47	A_130
Cyclohexanone	Ketones	40	A_31
Acetic acid ethyl ester	Acetate	39	A_239
Systolic blood pressure		31	
Heptane	Alkane	26	A_134
Acetophenone	Ketones	18	A_35
m-Xylene	Aromatics	16	A_222
Ethylbenzene	Aromatics	15	A_215
1,2-Dichloro-ethane	Halo-Hydrocarbons	13	A_93
Decane	Alkane	13	A_152
3-Heptanone	Ketones	13	A_25

Undecane	Alkane	12	A_154
Trichloroethene	Halo-Hydrocarbons	11	A_100
Phenol	Alcohol	11	A_62
Octadecane	Alkane	10	A_177
n-Nonane	Alkane	9	A_147
Sevoflurane	Other	9	A_299
Nonadecane	Alkane	8	A_178
Dihydro-2(3H)-furanone	Ketones	6	A_27
2-Ethyl-1-Hexanol	Alcohol	6	A_67
2-Methyl heptane	Alkane	5	A_141
2-Pentanone	Ketones	5	A_28
Benzaldehyde	Aldehyde	5	A_279
Octanal	Aldehyde	5	A_281
Sulfur dioxide	VSC	5	A_4

3-Methyl nonane	Alkane	5	A_121
Isoprene	Terpenes	4	A_187
Dimethyl selenide	Other	4	A_285
2-Pinene	Terpenes	4	A_198
Octane	Alkane	3	A_142
Myrcene	Terpenes	3	A_196
(-)-beta-Pinene	Terpenes	3	A_203
Toluene	Aromatics	3	A_213
o-Xylene	Aromatics	3	A_221
2,6-Dimethyloctane	Alkane	2	A_150
2,6-Dimethyloctane	Alkane	2	A_150
Allyl methyl disulfide	VSC	2	A_17
p-Xylene	Aromatics	2	A_219
Azulene	Aromatics	2	A_228

3-Sulfo-L-alanine	VSC	2	A_20
1,3,5-Trimethylbenzene	Aromatics	1	A_209
Propylcyclopentane	Alkane	1	A_138
Ethylcyclohexane	Alkane	1	A_139
Pentadecane	Alkane	1	A_169
(2E)-2-butenyl methyl sulfide	VSC	1	A_16
Acetone	Ketones	1	A_21
3-Carene	Terpenes	1	A_197
2-Butanone	Ketones	1	A_23
Phthalic anhydride	Aromatics	1	A_233
Acetic acid	Acid	1	A_260
Acetaldehyde	Aldehyde	1	A_273
Menthone	Ketones	1	A_40
Ethanol	Alcohol	1	A_46
